# Etude morphométrique de l'oreille externe chez les jeunes adultes

**DOI:** 10.11604/pamj.2014.19.355.4632

**Published:** 2014-12-05

**Authors:** Philippe Manyacka Ma Nyemb, Anne-Aurore Sankale, Lamine Ndiaye, Aïnina NDiaye, Magaye Gaye

**Affiliations:** 1 Laboratoire d'Anatomie, Unité de Formation et de Recherche des Sciences de la Santé, Université Gaston Berger, Saint-Louis, Sénégal; 2 Service de Chirurgie Générale, Centre Hospitalier Régional, Saint-Louis, Sénégal; 3 Service de Chirurgie Plastique, Esthétique et Reconstructrice, Hôpital Aristide LeDantec, Dakar, Sénégal; 4 Laboratoire d'Anatomie et d'Organogenèse, Faculté de Médecine, Université Cheikh Anta Diop de Dakar, Sénégal

**Keywords:** Oreille externe, morphométrie, adultes jeunes, outer ear, morphometry, young adults

## Abstract

**Introduction:**

Connaitre les dimensions normales de l'oreille externe constitue un pré-requis en chirurgie. Le but de ce travail est de déterminer les valeurs moyennes des différentes mesures morphométriques des oreilles gauches et droites dans notre population d’étude.

**Méthodes:**

Notre groupe d’étude était constitué de 100 sujets adultes jeunes (50 femmes et 50 hommes) âgés de 18 à 25 ans, et exempts de traumatismes ou d'anomalies congénitale de l'oreille externe. Les différentes mesures répertoriées étaient représentées par: la hauteur totale de l'oreille, la largeur totale de l'oreille, la hauteur lobulaire, la largeur lobulaire, la distance tragus - anti-hélix, la distance tragus - hélix, ainsi que la projection de l'oreille.

**Résultats:**

L’âge moyen de notre population d’étude était de 22 ans. La hauteur totale de l'oreille était respectivement de 61,6 et 60,3 mm chez les sujets de sexe masculin et féminin. La largeur totale retrouvée était respectivement de 32,5 et 30,8 mm. Pour toutes les distances mesurées, nous retrouvions des différences entre hommes et femmes, ainsi que d'un coté à l'autre.

**Conclusion:**

Pour le clinicien, il est important de prendre en compte les mesures morphométriques de l'oreille externe non seulement dans un but diagnostic, mais également pour reproduire lors de sa reconstruction une oreille anatomiquement correcte.

## Introduction

La connaissance des dimensions normales de l'oreille externe est indispensable pour le diagnostic de certaines malformations congénitales. Elle constitue également un pré-requis pour planifier la prise en charge de difformités acquises telles que les chéloïdes les séquelles de brûlures et les traumatismes de l'oreille externe, fréquentes dans notre contexte [1]. Les dimensions de l'oreille externe peuvent varier selon la race, le sexe, l’âge et même coté gauche ou droit. Le but de cette étude est de déterminer les valeurs moyennes des différentes mesures morphométriques des oreilles gauches et droites.

## Méthodes

**Figure 1:**
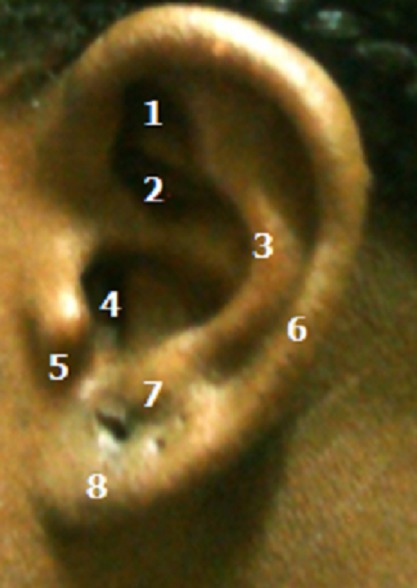
Anatomie de l'oreille normale

**Figure 2:**
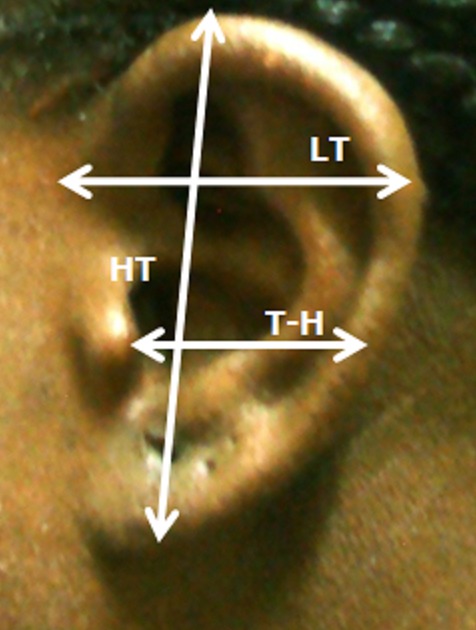
Mesures morphométriques de la hauteur totale (HT), la largeur totale (LT) et la distance tragus – hélix (T-H)

**Figure 3:**
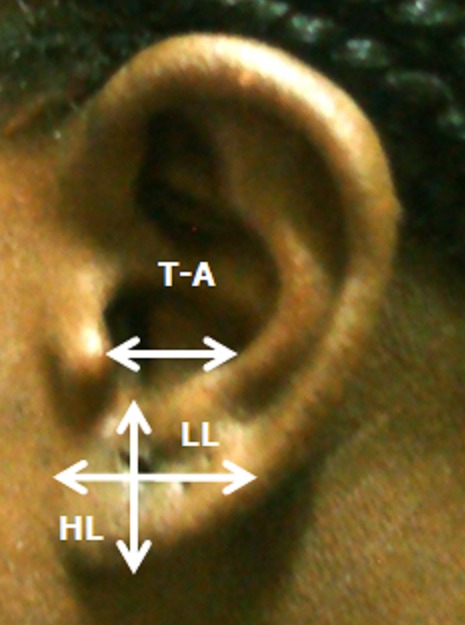
Mesures morphométriques de la hauteur lobulaire (HL), la largeur lobulaire (LL) et la distance tragus – antihélix (T-A)

Notre groupe d’étude était constitué de 100 sujets adultes jeunes (50 femmes et 50 hommes) âgés de 18 à 25 ans, et exempts de traumatismes ou d'anomalies congénitale de l'oreille externe (Figure 1). Les différentes mesures ont été effectuées au pied à coulisse, et les données ont été exploitées à l'aide du logiciel SPSS version 18. Les mesures effectuées étaient représentées par les distances suivantes (Figure 2 et Figure 3): La hauteur totale de l'oreille (HT): distance séparant le sommet de l'hélix du point le plus déclive du lobule; La hauteur lobulaire (HL): distance entre l'incisure intertragique et l'extrémité caudale du lobule; La largeur lobulaire (LL): représentée par la largeur horizontale du lobule à mi-distance de la hauteur lobulaire; La distance séparant le tragus de l'antihélix (T-A); La distance séparant le tragus de l'hélix (T-H); La projection de l'oreille: distance séparant l'hélix du processus mastoïde à hauteur du tragus (Pm-H); La largeur totale de l'oreille (LT): distance entre le point le plus antérieur et le point le plus postérieur de l'oreille externe.

## Résultats

L’âge moyen de notre population d’étude était de 22 ans, et le sex-ratio de 1. Les résultats détaillés sont représentés dans le Tableau 1.

**Tableau 1:** Mesures morphométriques réalisées chez 50 hommes et 50 femmes

Distances mesurées	Sexe	Coté	Moyennes (en millimètres)	Déviations standard (en millimètres)
Hauteur totale de l'oreille (HT)
	Masculin	Gauche	61,6	3,3
		Droit	61,4	3,4
	Féminin	Gauche	60,3	3,0
		Droit	60,1	2,9
Hauteur lobulaire (HL)
	Masculin	Gauche	18,1	1,6
		Droit	18,3	1,5
	Féminin	Gauche	17,2	1,3
		Droit	17,6	1,4
Largeur lobulaire (LL)
	Masculin	Gauche	19,2	2,0
		Droit	19,5	2,1
	Féminin	Gauche	18,6	2,1
		Droit	18,2	2,1
Distance du tragus à l'antihélix (T-A)
	Masculin	Gauche	17,5	1,6
		Droit	17,4	1,7
	Féminin	Gauche	16,7	1,6
		Droit	16,8	1,6
Distance du tragus à l'hélix (T-H)
	Masculin	Gauche	26,7	2,1
		Droit	26,4	2,0
	Féminin	Gauche	25,2	2,0
		Droit	25,0	1,9
Projection de l'oreille ou distance processus mastoïde-hélix (Pm-H)
	Masculin	Gauche	17,4	2,1
		Droit	17,5	2,1
	Féminin	Gauche	16,2	1,9
		Droit	16,1	1,9
Largeur totale de l'oreille (LT)
	Masculin	Gauche	32,5	2,0
		Droit	32,3	2,2
	Féminin	Gauche	30,5	2,2
		Droit	30,5	2,1

## Discussion

La connaissance de l'anatomie de l'oreille normale est importante. Elle permet au chirurgien plasticien de planifier le traitement chirurgical des malformations de l'oreille externe. D'ailleurs lors de sa reconstruction esthétique, l'objectif du chirurgien est de conférer à l'oreille externe l'apparence la plus jeune possible [2]. C'est pour cette raison que notre population d’étude est composée d'adultes jeunes dont l’âge varie entre 18 et 25 ans. L'anatomie de l'oreille est également importante pour le calibrage des appareils d'audition. En outre, l'oreille humaine fait partie des caractéristiques esthétiques qui définissent le visage, son apparence et sa symétrie sont importants pour l'harmonie faciale. Sa configuration serait liée à l’âge et au sexe des individus [2]. Les anomalies des oreilles telles que la ptose du lobule, l'agénésie de l'oreille externe, la microtie ou les oreilles proéminentes peuvent résulter d'un traumatisme, d'une résection chirurgicale, de tumeurs ou de malformations congénitales [3]. Dans notre contexte, les malformations acquises sont principalement dues aux chéloïdes, aux séquelles de brulures et aux traumatismes de l'oreille. De nombreuses études ont été faites concernant les anomalies de l'oreille externe, mais les dimensions et la forme de cet organe n'ont été que peu étudiés dans la population normale [4, 5]. L'oreille externe est constituée de 3 composants essentiels: le complex hélix-antihélix, le complex conchal et le lobule (Figure 1).

La hauteur totale de l'oreille est importante pour l’évaluation des anomalies congénitales telles que le syndrome de Down [6]. Dans une population nord-américaine de race blanche, la hauteur totale retrouvée est respectivement de 62,4 mm et 58,5 mm chez l'homme et chez la femme [6]. Plus récemment, une étude réalisée en Turquie [7] retrouve des valeurs de 63,1 et 59,7 mm. Notre travail rapporte des dimensions similaires, avec en moyenne 61,5 mm chez l'homme et 60,2 mm chez la femme. Malgré des particularités anatomiques propres à chaque race, le travail d'Adamson portant sur 2300 oreilles montre qu'il n'existe aucune différence sur la croissance de l'oreille externe entre les diverses races telles que les blancs et les noirs [8]. Concernant les dimensions du lobule, Bozkir [7] retrouve une hauteur lobulaire de 18,4 mm chez l'homme et 17,7 mm chez la femme. Nous retrouvons respectivement des valeurs de 18,2 mm et 17,4 mm. Dans le travail de Brucker et al [2], la largeur du lobule est de 19,5 mm chez l'homme et 19,7 mm chez la femme. Bozkir retrouve respectivement 19,4 et 18,4 mm [7]. Notre travail rapporte respectivement des valeurs semblables de 19,3 et 18,4 mm.

Les distances tragus - hélix et tragus - antihélix sont essentielles pour le diagnostic des malformations auriculaires. Ces distances sont également utilisées pour la fabrication et le calibrage du matériel d'audition. Dans son travail, Bozkir retrouve des distances tragus - hélix et tragus - antihélix de 26,3 et 17,2 mm chez l'homme et de 25,1 et 16,6 mm chez la femme [7]. Dans notre étude, ces mêmes distances sont de 26,5 et 17,4 mm chez l'homme, et de 25,1 et 16,7 mm chez la femme. Dans la littérature, la projection de l'oreille varie généralement entre 15 et 20 mm [9]. Cet intervalle est concordant avec nos résultats, avec des mesures de 17,4 mm chez l'homme et 16,1 mm chez la femme. Dans un échantillon constitué de 100 hommes et 100 femmes [10], la largeur de l'oreille retrouvée chez l'homme est de 32,4 mm pour l'oreille gauche et 33 mm pour l'oreille droite. Chez la femme les valeurs sont de 31,9 mm à gauche et 32,4 mm à droite. Dans notre travail, nous retrouvons également des différences non significatives entre les oreilles gauche et droite. Plusieurs travaux montrent qu'il existe des différences entre les dimensions des moitiés gauche et droite de la face [11]. Ces différences sont visibles au niveau des structures paires telles que les yeux, les oreilles ou les joues. Après analyse de ses résultats, Bozkir retrouve que chez l'homme la hauteur totale et la largeur totale sont plus importants [7]. Brucker conclut qu'il retrouve une différence des mesures morphométriques de l'oreille pouvant atteindre 6,5% d'un sexe à l'autre [2]. Mais très souvent elles sont non significatives. Dans notre travail, des différences non significatives sont retrouvées concernant la largeur totale, la projection de l'oreille, ainsi que la largeur et la hauteur lobulaire. Ceci suggère que le port de la boucle d'oreille ne constitue qu'un facteur parmi d'autres pouvant influencer les dimensions du lobule.

Il est probable que l'influence du port de la boucle d'oreille soit plus visible chez des sujets plus âgés. Brucker [2] retrouve cette tendance dans son travail. Dans le même sens, Adamson et al. Suggèrent que 85% de la croissance de l'oreille se produit avant l’âge de 3 ans, et que les 15% restant se produisent avant l’âge de 20 ans [8]. Ainsi, l'augmentation en taille observée au-delà de 20 ans serait attribuable à une élongation du lobule de l'oreille secondaire aux forces gravitationnelles telles que celle observées lors du port de la boucle d'oreille. Dans notre travail, nous n'avons pas étudié l'influence de l’âge sur les dimensions et la configuration du lobule. Dans son travail sur la croissance lobulaire, Azaria [12] va plus loin en expliquant que le lobule continue de croître bien après l’âge de 20 ans. Ainsi, la longueur du lobule de l'oreille augmenterait de 30 à 35% entre un groupe âgé de 20 à 40 ans, et un groupe âgé de plus de 60 ans. Cette augmentation serait due à un excès cutané et à la diminution des forces de traction dans le tissu conjonctif avec le temps. Comme dans notre travail et comme dans l’étude de Ferrario [11], Azaria retrouve que dans la population générale, les lobules de l'oreille ne sont pas aussi symétriques qu'on pourrait s'y attendre [12]. Le lobule gauche est retrouvé en moyenne plus court que le lobule droit, et cette disparité est fréquente. Notre travail montre qu'il existe des similitudes dans la morphométrie de l'oreille externe, y compris entre des populations de race différente. Cependant il ressort de cette étude quelques particularités morphométriques, notamment en ce qui concerne les dimensions du lobule. Un échantillon plus représentatif que le notre permettrait probablement de documenter cette hypothèse.

## Conclusion

Ce travail rapporte les valeurs moyennes des différentes mesures morphométriques de l'oreille externe. Il est important de prendre en compte ces dimensions afin de déterminer d’éventuelles variations anatomiques ou anomalies congénitales. Elles permettent également au clinicien de reproduire lors de sa reconstruction une oreille anatomiquement correcte.
